# Public policies for the promotion of paralympic sport in Latin America: a comparative analysis of 10 countries

**DOI:** 10.3389/fspor.2026.1844536

**Published:** 2026-06-08

**Authors:** Beatriz Revuelta, Raynier Hernández

**Affiliations:** 1 Universidad Central de Chile, Santiago, Chile; 2 University of the Americas, Santiago, Chile

**Keywords:** ableism, disability, legislation, paralympic sport, public policy, Latin America, comparative analysis

## Abstract

**Introduction:**

This article comparatively analyzes public policies for the promotion of Paralympic sport in ten Latin American countries with outstanding results in the Paralympic medal table during the last Olympic cycle: Brazil, Colombia, Mexico, Argentina, Cuba, Venezuela, Costa Rica, Chile, Peru, and Ecuador. From a social study of disability perspective, it examines how these countries have incorporated sport practiced by persons with disabilities into their regulatory frameworks, what support instruments they have developed, and to what extent they have built institutional mechanisms to foster access, retention, and athletic development.

**Methods:**

The study is based on a comparative documentary analysis of laws, regulations, programs, institutional plans, and official documents.

**Results:**

The results show that most countries have advanced formal recognition of the right to sport for persons with disabilities and in the creation of support instruments, especially scholarships and awards associated with high performance. However, these mechanisms are mainly concentrated on rewarding achievements already attained rather than correcting structural inequalities in access. The main gaps appear in talent identification, in the articulation of pathways for athletic progression, and in the incorporation of gender- and territory-based approaches.

**Discussion:**

The article concludes that the region has made greater progress in normative recognition and performance incentives than in consolidating comprehensive, accessible, and equitable systems. The main public policy challenge is to strengthen stable conditions for the training, continuity, and development of athletic trajectories.

## 1 Introduction

In recent decades, Paralympic sport has acquired growing centrality as an object of public policy, sport governance, and rights recognition. This shift expresses a broader transformation in ways of understanding disability: from predominantly biomedical and assistance-based approaches centered on individual impairment toward perspectives that emphasize the removal of barriers. Within this framework, the Convention on the Rights of Persons with Disabilities (1) constitutes a decisive normative milestone, insofar as Article 30 enshrines the right of persons with disabilities to participate, on an equal basis with others, in recreational, leisure, and sporting activities. At the same time, the consolidation of the Paralympic Movement has given rise to a highly specialized sporting field regulated by international standards, in which sport-specific classification has become a central mechanism for the technical validation of competition and for the institutional legitimacy of the system in each country. However, legal recognition of the right to sport does not guarantee its effective realization. Recent research shows that the robustness of public policy in this area depends on its translation into stable institutional arrangements, sustained funding mechanisms, accessible infrastructure, specialized technical support, and organizational devices capable of accompanying sport trajectories over time (2–4). In other words, the effective exercise of the right to sport requires more than its normative inscription: it presupposes the existence of material, administrative, and technical conditions that make it possible for persons with disabilities to access, remain in, and progress within the sport system.

From this perspective, Paralympic sport constitutes an analytically privileged terrain for examining the relationship between formal recognition, state capacity, and substantive equality. Within this framework, functional classification occupies a particularly relevant place. Far from being a merely technical procedure, classification organizes eligibility, distributes competitive legitimacy, and structures the confidence of athletes, coaches, and institutions in the integrity of the Paralympic system. For this reason, different studies have emphasized that classification based on robust evidence and transparent criteria constitutes an indispensable condition for ensuring comparability and competitive fairness (5, 6). Added to this dimension are other factors that are equally critical to the sustainability of sport trajectories, such as the availability of multidisciplinary teams, the stability of monetary and non-monetary support, the administrative burden of eligibility processes, the predictability of calendars, and the existence of adequate resources for athletes' well-being and mental health (7, 8). In this sense, Paralympic performance cannot be interpreted as the exclusive result of individual effort, but rather as the effect of an institutional architecture that distributes opportunities, resources, and conditions of continuity unequally.

Latin America offers a particularly interesting setting in which to examine advances or gaps (9, 10). The region combines important normative advances in recognizing the sport of persons with disabilities with marked heterogeneities in the density of its operational instruments, the formalization of its support mechanisms, and the capacity of its sport systems to structure sustainable trajectories (10, 11). Whereas some countries have developed relatively articulated arrangements in financing, scholarships, non-monetary support, talent identification, and progression toward high performance, others display more fragmented devices that are weakly protocolized or only faintly visible in public documentation (9, 12–16). This diversity not only reflects different degrees of institutional development, but also different ways of conceiving the relationship between disability, sport, and state action. For that reason, comparative analysis of the region makes it possible to question not only which policies exist, but also how they operate, which inequalities they correct, and which they reproduce. From the standpoint of the social studies of disability, these questions are especially pertinent (17–19). A significant part of the critical literature has warned that Paralympic sport can function simultaneously as a space of recognition and as a mechanism for reproducing hierarchies, insofar as inclusion is often organized around heroic, meritocratic, and exceptional narratives (20, 21). Under this logic, the athlete with a disability tends to be recognized in terms of the ability to adapt to standards of performance, productivity, and excellence historically defined from ableist matrices (18, 22).

Consequently, public policy risks concentrating on rewarding already-consolidated achievement rather than intervening in the structural conditions that make access to practice, continuity of trajectories, and competitive projection possible or impossible (23–25). Analyzing policies for the promotion of Paralympic sport from this perspective therefore requires shifting attention away from the isolated sport result and toward the institutional mediations that condition participation and retention. On this basis, this article comparatively analyzes public policies for the promotion of Paralympic sport in the ten Latin American countries with the best recent performance in the Paralympic medal table: Brazil, Colombia, Mexico, Argentina, Cuba, Venezuela, Costa Rica, Chile, Peru, and Ecuador.

The study examines three major dimensions: the normative frameworks that recognize and regulate the sport of persons with disabilities; the operational devices, including economic incentives, non-monetary support, infrastructure, and technical-sport mechanisms; and the components of governance and equity, with special attention to talent identification, the transition between school, club, and national team, and gender and territorial inequalities. The objective is to identify convergences, differences, and gaps in the regional architecture for the promotion of Paralympic sport, attending not only to the existence of instruments, but also to their degree of articulation, formalization, and redistributive potential. The central argument guiding this work is that the consolidation of Paralympic sport in Latin America cannot be assessed exclusively on the basis of the volume of incentives or performance in the medal table. Rather, it must be analyzed in terms of the capacity of states to build accessible, continuous, transparent, and equitable systems of training, support, and sport progression. In this sense, this work offers a comparative systematization of public policy instruments and devices in the region that has not previously been produced, and it proposes a reading of Paralympic sport as a field traversed by tensions among formal recognition and effective implementation, merit, equality, ableism, high performance, and sport justice that cannot be understood without the definitions of disability that underlie different countries.

## 2 Research on paralympic sport policy, governance, and sport justice

The Convention on the Rights of Persons with Disabilities (1) consolidated a fundamental normative standard by recognizing the right of persons with disabilities to participate, on an equal basis, in recreational and sporting activities. However, the recent literature agrees that legal recognition alone does not guarantee the effective exercise of that right (2–4). From the social studies of disability, this tension can be understood as a gap between formal recognition and material equality. The social model showed early on that exclusion derives not only from bodily impairment, but also from social, institutional, and cultural barriers that restrict participation (26, 27, 34). In line, critical perspectives have warned that those barriers operate through concrete procedures of classification, access, representation, and institutional validation, which distribute resources, legitimacy, and opportunities unequally (19, 28, 33). Consequently, the analysis of Paralympic sport requires questioning not only the existence of norms or programs, but also the real capacity of sport systems to produce conditions of access, continuity, and progression in non-ableist terms. This perspective is especially relevant in contexts where inclusion is often organized around the exceptionality of achievement and not necessarily around the redistribution of the conditions that make it possible. The literature on comparative sport policy also shows that Paralympic performance does not depend exclusively on aggregate structural variables, but on the coherence of the institutional framework that sustains competitive practice. The countries with the best performance tend to combine multi-year planning, stable incentives, and robust insertion of Paralympic committees in sport governance (2).

Within national sport systems, National Paralympic Committees (NPCs; CPN in Spanish) typically operate as key governance and coordination actors for Paralympic sport. They often interface with public sport authorities and sport-specific federations in areas such as athlete pathways, selection processes, and the organization of participation in elite parasport structures. In comparative policy terms, the formal role assigned to NPCs/CPN in national instruments is analytically relevant because it signals how Paralympic sport governance is institutionalized and implemented, and how responsibilities are distributed across actors (2, 24).

In a convergent sense, the studies emphasize that the quality of public policy can be evaluated more precisely when verifiable criteria are observed, such as eligibility, talent identification calendars, evaluation protocols, and follow-up mechanisms (3, 23). In this way, the analytical focus shifts from the existence of state support to the operational quality of the system, which is a key issue for analyzing policies in contexts of institutional heterogeneity such as those in Latin America. Added to this is the need to understand high-performance Paralympic sport as a trajectory sustained by support networks rather than as a simple expression of individual merit. Alexander et al. (8) show that support teams are traversed by tensions linked to administrative overload, uncertainty in calendars, and deficits in interinstitutional coordination (8). In parallel, Cosh et al. (7) point out that access to psychological support remains limited, revealing the persistence of cultural and organizational barriers in the field of mental health. These findings broaden the notion of sport policy: it is not only about scholarships, medals, or training, but also about well-being, care, and institutional stability (7).

In the case of Paralympic sport, this observation is especially relevant, given that competitive continuity often depends on additional mediations such as functional classification, human support, specialized equipment, and care arrangements that are rarely visible in narratives centered exclusively on performance. It has also been observed that Paralympic sport continues to be traversed by heroic and meritocratic narratives that exalt individual resilience and represent the athlete with a disability as an exceptional figure (29–31). Although these narratives can increase visibility, they also risk exoticizing differences and obscuring the structural conditions that sustain or restrict performance. By contrast, Matson-Barkat et al. (32) suggest that certain forms of public communication can contribute to destigmatization when they shift the focus from deficit toward athletic performance in the fullest sense (32). In Garland-Thomson's terms (2011), public policy also organizes regimes of visibility that define which bodies are intelligible, admirable, or worthy of support. From this perspective, analyzing Paralympic policies also entails examining the symbolic frameworks through which disability is represented, and sporting excellence is legitimized.

## 3 Methods

This study uses a comparative documentary analysis to examine the normative and programmatic scaffolding of Paralympic sport across ten Latin American countries with sustained participation and strong performance in the Tokyo 2020–Paris 2024 cycle (Brazil, Colombia, Mexico, Argentina, Cuba, Venezuela, Costa Rica, Chile, Peru, and Ecuador). Country selection followed a transparent, cycle-specific performance criterion: we included the ten Latin American countries with the highest total medal counts across the Tokyo 2020 and Paris 2024 Paralympic Games, based on the official International Paralympic Committee (IPC) medal tables. Countries outside this set were excluded because they did not rank among the top ten under the same Tokyo–Paris medal-based rule.

The methodological objective was to characterize and compare how national public policies: (i) recognize the right to sport for persons with disabilities; (ii) define and regulate adaptive/Paralympic sport; (iii) establish governance arrangements for Paralympic sport (including the role of National Paralympic Committees and sport-specific federations); and (iv) translate these commitments into operational instruments. We focused on policy features that shape athlete development and professionalization, including technical-sport support (sport-specific classification and eligibility) and pipeline mechanisms (talent identification and school–club–federation progression), as well as accessibility and equity. CRPD ratification is reported as a contextual benchmark for rights-based policy alignment; the analysis does not assume simultaneous adoption or direct causality from ratification to specific national instruments.

The observation window for document retrieval was 2000–2025, including earlier instruments when they remained in force or retained transitional effects. The final documentary corpus used to produce Tables 2–7 comprised *N* = 45 official documents. The number of documents per country ranged from 1 to 7, and the publication-year coverage spans 1974–2025. Table 1 reports the number of documents by country and the publication-year range.

**Table 1:** Documentary corpus: number of documents by country and publication-year range.

Country	Documents analyzed (n)	Publication years covered (range)
Brazil	4	2004–2025
Colombia	7	2000–2022
Mexico	6	2011–2024
Argentina	5	1974–2014
Cuba	2	1976–2025
Venezuela	2	2006–2011
Costa Rica	6	1996–2024
Chile	5	2001–2025
Peru	4	2003–2020
Ecuador	4	2010–2025
Total	45	1974–2025

**Table 2:** Analytic dimensions and indicators for the comparative documentary analysis.

Dimension	Core indicators
Legal guarantees	Explicit right to sport for persons with disabilities; legal definition of adaptive and/or Paralympic sport; explicit rights-based obligations
Institutional arrangements	Formal recognition and functions of the National Paralympic Committee; defined coordination with ministries and sport-specific federations
Policy instruments	Scholarship/incentive regulations; non-monetary supports (e.g., training services, insurance, travel support when formalized); tax incentives or national plans with identifiable implementation mechanisms
Technical-sport support	Publicly documented classification and eligibility provisions (rules or guidance); procedures and/or calendars; designated institutional responsibility for classification/eligibility processes
Pipeline (talent identification and progression)	Documented talent identification mechanisms; defined responsibilities or stages for progression (school–club–federation/national team); criteria or procedures for advancement when specified
Equity and accessibility	Accessibility requirements applicable to sport settings; gender- and/or territory-sensitive measures expressed as targets, operational requirements, or monitoring/reporting obligations

**Table 3:** Definitions of disability and inclusion of paralympic sport by country.

Country	Laws	Definitions concerning disability and sport
Brazil	Brazilian Inclusion Law (LBI) Law No. 13,146 (2015)	It adopts a definition of a person with a disability based on long-term impairments that, in interaction with barriers, restrict social participation; it also expressly recognizes the right to sport on an equal basis. The LBI declares the CRPD as a foundation, and the normative design of sport is complemented by the Sports Incentive Law and by the institutional architecture of the Ministry of Sport regarding parasport, showing alignment with the international standard of rights and accessibility.
Colombia	Statutory Law 1618 (2013) Law 582 (2000) (Law 1946)	It ensures the full exercise of the rights of persons with disabilities and mandates affirmative actions that include strengthening Paralympic sport, with guarantees for training, medical support, and functional classification within the National Sport System. Law 582 (2000), in turn, early on defined the “associated sport of persons with disabilities,” and the 2019 governance reform (Law 1946) shifted the axis from ‘types of disability’ toward sport-specific governance, bringing the national structure closer to the international governance logic by sport.
Mexico	General Law for the Inclusion of Persons with Disabilities General Law on Physical Culture and Sport	It recognizes rights and state obligations from a human-rights perspective, while the General Law on Physical Culture and Sport prohibits discrimination and establishes sport as a fundamental right for all persons, including athletes with disabilities. Mexico has also incorporated a health standard (NOM-039-SSA-2023) to certify disability under a biopsychosocial model aligned with the CRPD and the International Classification of Functioning, Disability and Health (ICF), a relevant technical connection for the sport eligibility and classification required by the IPC.
Argentina	Law 22,431 Law 27,044 Sports Law (20,655) Law 26,573	Law 22,431 incorporates a definition of disability aligned with the Convention on the Rights of Persons with Disabilities, while Law 27,044 grants constitutional hierarchy to that Convention. In sport matters, Law 20,655 recognizes sport and physical activity as rights of the population, provides special opportunities for persons with disabilities, and contemplates adapted sport within the institutional system. Specific support for high-performance Paralympic sport is also articulated through ENARD, created by Law 26,573.
Cuba	Law on the Cuban Sport System (2025)	It declares an inclusive and rights-guaranteeing system, recognizes the universal character of the right to sport, and orders the structure and functions within which the Cuban Paralympic Committee is inserted. Although the 2025 legal text does not develop its own detailed definition of disability—which in practice has been interpreted in accordance with the Constitution and international instruments—it does establish an explicit framework of inclusion for sport and recognition of the Paralympic movement. The link to IPC classification standards exists indirectly, through technical and federative implementation rather than as an express mandate of the legal text.
Venezuela	Law for Persons with Disabilities (2006) Organic Law on Sport (2011)	The Law for Persons with Disabilities (2006) defines disability through a mixed formulation, with biopsychosocial language and interaction with barriers, although it maintains a functional enumeration of temporary or permanent impairments and limitations. The law itself recognizes principles of equality and non-discrimination and mandates public policies for the inclusion of persons with disabilities in sport practice. The Organic Law on Sport, Physical Activity, and Physical Education (2011), for its part, establishes sport as a fundamental right and public service, expressly incorporates Paralympic athletes within its definitions, and recognizes the Venezuelan Paralympic Committee as an actor within the sport system. Its articulation with the CRPD and with IPC sport-specific classification should be understood mainly in interpretive and regulatory terms rather than as an express mandate of these laws.
Costa Rica	Law 7600, with definitional reform of 2014	Its definition of disability moved toward the interaction model, considering long-term impairments and environmental barriers, and is aligned with the CRPD; it also recognizes access to culture, recreation, and sport. Law 9739 (2019) reformed the system to explicitly include sport and recreation for persons with disabilities and, with respect to the National Paralympic Committee, expressly recognizes it, grants it its own legal personality, and regulates its powers, its relationship with the Institute, and its national and international representation in the Paralympic cycle. Both Law 7600 and its regulations also classify denial of sport participation on grounds of disability as a discriminatory act.
Chile	Law 20,422	It adopts a biopsychosocial definition in line with the CRPD. The Sport Law (19,712) incorporated the notion of adapted sport, defining it in a way compatible with Paralympic practice and the preservation of the ‘essence of sport.’ The Chilean normative framework explicitly links the right to sport, accessibility, and universal design to participation in the sport system; consequently, national implementation can align with the IPC classification regime through the federations of each parasport.
Peru	Law 29973 (2012)	It defines a person with a disability through the interaction model between permanent impairments and barriers that restrict the exercise of rights. It devotes a specific chapter to sport that establishes the role of the IPD in promoting participation and recognizes federations of persons with disabilities. This architecture refers directly to Article 30 of the CRPD and facilitates adoption of IPC sport-specific classification in coordination with federative entities and Regional Sport Councils. Nevertheless, the law does not expressly regulate adapted or Paralympic sport clubs nor does it directly develop IPC classification standards, which operate mainly at the regulatory and federative level.
Ecuador	Organic Law on Disabilities (2016) Organic Law on Persons with Disabilities (2025) Law on Sport, Physical Education, and Recreation (2010)	From its purpose onward, it incorporates the enforceability of rights established in the Constitution and in international treaties, and its updated version (2025) expressly regulates adapted or Paralympic sport clubs and the promotion of the right to sport and recreation for persons with disabilities, consolidating an explicit anchoring in the CRPD. Sectoral regulations and complementary documents also recognize the role of CONADIS and coordination with the governing body, creating a bridge to IPC classification standards in each discipline. Institutional coordination with the National Council for the Equality of Disabilities and the sport governing body strengthens system implementation, while articulation with IPC standards operates mainly through the sport regime and the Ecuadorian Paralympic Committee, rather than as an explicit rule of classification in disability law.

**Table 4:** High-performance scholarship and incentive programs covering paralympic athletes by country.

Country	Name of scholarship/program	Who can access it?	Conditions of the scholarship or support
Brazil	Bolsa Atleta (including podium category)	High-performance athletes, including para-athletes; in the podium category, athletes ranked among the best in the world.	Monthly scholarship organized by performance categories: base, student, national, international, Olympic/Paralympic/Deaflympic, and podium. Access depends on sport results, ranking position, competitive trajectory, and recognition within the national high-performance system.
Colombia	Ministry of Sport financial support (Podium/Excellence Athlete programs)	Athletes in the high-performance system, including para-athletes linked to ministerial programs.	Access depends on sport level, results obtained, and linkage to the national sport system. Support is assigned according to criteria of performance, projection, and competitive continuity.
Mexico	CONADE scholarships (Physical Culture and Sport Program)	Athletes in conventional and adapted sport incorporated into processes recognized by CONADE.	Financial support is assigned to athletes with results in official competitions and continuity in selection and high-performance processes. Continuity depends on performance, institutional evaluation, and the current operating rules.
Argentina	ENARD Scholarship System	High-performance athletes, including athletes and para-athletes, with officially recognized sporting merit and achievements.	Monthly scholarships granted according to sport achievements, participation in relevant competitions, and criteria established by the current ENARD regulations.
Cuba	Basic monthly income for high-performance athletes	Athletes integrated into the state high-performance system of national preselection squads.	Access depends on formal incorporation into the national sport system and institutional recognition of the athlete within the state structure of competitive sport.
Venezuela	Simón Bolívar Sport Scholarship System	Athletes, para-athletes, and coaches linked to the national sport system.	Financial support is assigned according to competitive level, results obtained, and recognition of the athlete by sport institutions.
Costa Rica	Incentives for High-Level Athletes (ICODER)	High-level athletes, including Paralympic athletes.	Access depends on sport results, competitive level, regulatory score/categorization, and recognition as a high-level athlete within the system administered by ICODER.
Chile	PRODDAR Scholarship (Scholarship System for High-Performance Athletes)	High-performance athletes, including Paralympic athletes and, in certain cases, their guides or support operators.	Monthly stipend assigned according to sport achievements, category, and continuity in the program. In the case of Paralympic sport, it also recognizes guides in certain disciplines in accordance with the current regulations.
Peru	Athlete Support Program (PAD)	Qualified Athletes (DC) and High-Level Qualified Athletes (DCAN), including para-athletes recognized by the IPD.	Monthly subsidy scaled according to category, performance, and results in official competitions. Access depends on the institutional recognition granted by the IPD and on the athlete's position within the support system.
Ecuador	High Performance Plan (2025–2028 cycle/Los Angeles 2028)	High-performance athletes, including para-athletes, classified in competitive categories.	Monthly financial incentive defined according to sport category, results, and competitive trajectory. Access depends on formal inclusion in the current High Performance Plan.

**Table 5:** Non-monetary support by country.

Country	Non-monetary support
Brazil	Access to centers and projects financed through the Lei de Incentivo ao Esporte; articulation through the university network and the national Paralympic system.
Colombia	System services such as functional classification, technical support, and access to facilities of the National Sport System, coordinated with the Colombian Paralympic Committee.
Mexico	Access to the Mexican Paralympic Center (CEPAR) and to sports medicine and applied sport science services through CONADE.
Argentina	Coverage of multidisciplinary teams, travel, training camps, and equipment, in accordance with the annual sport preparation plan.
Cuba	Universal access to services, athletes’ rights and duties, facility management, and express recognition of the Cuban Paralympic Committee.
Venezuela	Access to system services, including sport facilities, events, and institutional recognition of the Venezuelan Paralympic Committee (CPV).
Costa Rica	Technical services of the system and articulation with the National Paralympic Committee within the institutional structure of high-level sport.
Chile	Supplementary insurance, maternity and child care support, access to training centers, and inclusion of assistance roles, as in boccia, within PRODDAR.
Peru	Private medical insurance, multidisciplinary teams, travel, and specialized preparation for sustaining the sport trajectory.
Ecuador	Medical insurance, specialized technical teams, follow-up, and prioritized access to services for sport preparation.

**Table 6:** Talent identification system by country.

Country	Talent identification
Brazil	Paradesporto Brasil em Rede (PPBR) creates university-based hubs for initiation and talent identification; it is articulated with the National Secretariat of Parasport (SNPAR).
Colombia	Law 1946 of 2019 reorganizes governance by sport and enables talent identification within each federation, no longer by type of disability; with subsequent regulation in 2021.
Mexico	CEPAR (Mexican Paralympic Center) functions as a technical hub for identification and development; COPAME publishes sport-specific classifications and team selections, while CONADE scholarships sustain the flow.
Argentina	ENARD operates a high-performance certification system with a network of evaluators and nationwide scouting; COPAR provides training for coaches and classifiers, strengthening the pipeline.
Cuba	The new Law on the Cuban Sport System (2025) recognizes and organizes the functions of the Cuban Paralympic Committee, but it does not yet detail a specific national identification program.
Venezuela	Communications from the Ministry of Sport and COPAVEN report training camps and pre-selections, but no structured and public national talent identification system appears.
Costa Rica	ICODER has regulations and selective programs; in addition, FODESAF evidence shows follow-up of delegations and sexes, although no single document of systematic identification for parasports has been identified.
Chile	Promesas Chile (IND) develops talent identification for athletes aged 9 to 21 for the Olympic and Paralympic cycle; it is complemented by camps with COPACHI to identify new prospects.
Peru	The IPD-PAD finances development and services for para-athletes; talent identification appears mentioned in amendments to Law 28036, but no specific public protocol for parasports is observed; sectoral initiatives exist.
Ecuador	The High Performance Plan (PAR 2022-2025) also identifies, categorizes, and selects para-athletes; the CPE publishes high-performance categories and quotas.

**Table 7:** School-club-national team transition.

Country	School-club-national team transition
Brazil	The transition is configured through a staged route of sport incorporation in which university hubs and initiation spaces serve an early identification and training function; athletes subsequently move on to clubs and federations, from which they may be integrated into the preparation circuits of the Brazilian Paralympic Committee. This mechanism shows a relatively structured articulation between the training base, technification, and national representation.
Colombia	The transition mechanism is organized through functional coordination among the Ministry of Sport, the Colombian Paralympic Committee, and sport-specific federations, allowing the athlete's trajectory to move from initial spaces of practice and training toward federated structures and, finally, toward the national team. Institutional reorganization by sport, rather than by type of disability, strengthens a logic of competitive specialization.
Mexico	In Mexico, the transition operates as an institutional chain connecting state and municipal programs with clubs, federations, and specialized high-performance devices, particularly the Mexican Paralympic Center and the Mexican Paralympic Committee. This route allows athletes to be progressively evaluated, brought into camps, and projected toward the national team through relatively differentiated technical instances.
Argentina	The Argentine mechanism is structured through articulation among clubs, federations, and national high-performance bodies, especially the National Entity for High-Performance Sport and the Argentine Paralympic Committee. Added to this is the intervention of the National Disability Agency in processes linked to training and classification, configuring a trajectory in which the transition to the elite depends on coordination between sport institutions and state agencies.
Cuba	The transition is embedded in a centralized state model in which the athletic trajectory unfolds from community and school spaces toward the structure of the National Institute of Sports, Physical Education, and Recreation. Although the system shows strong vertical organizational capacity, the available documentation does not allow the clear identification of specific and differentiated protocols for the Paralympic transition.
Venezuela	In Venezuela, the transition appears to operate through a general route of advancement from state associations and federations toward the national team, but without an updated public instrument that details its stages, criteria, or support mechanisms with precision. This suggests an existing institutional scheme, though one that is less formalized and less visible in the documentation.
Costa Rica	The Costa Rican mechanism is based on a progression from the cantonal level and clubs toward the National Paralympic Committee of Costa Rica, in articulation with scholarship and incentive regulations administered by the sport system. The route evidences a connection between the local and national competitive levels, although with less programmatic density than in other regional cases.
Chile	In Chile, the transition is organized through a progressive trajectory that begins in school and community spaces, continues through sport projection programs such as Promesas Chile and in clubs or federations, and culminates in the national team. This model suggests a relatively clear route of escalation in which early identification and intermediate development play an important role in consolidating Paralympic careers.
Peru	The Peruvian case shows a transition built through articulation among federative and regional programs, the Peruvian Institute of Sport, and the Athlete Support Program, with an important support point in South American school competitions. In this way, the trajectory combines grassroots spaces, institutional projection, and mechanisms for sustaining high performance.
Ecuador	In Ecuador, the transition is configured through an institutional sequence linking federations and the Ecuadorian Paralympic Committee with the High-Performance Plan, which classifies and organizes athletes according to results and competitive categories. This mechanism progressively integrates training, evaluation, and international projection within a state structure of financing and follow-up.

### 3.1 Document identification and selection

Document identification relied on official public sources and followed a structured search approach using Boolean logic (AND/OR operators) across predefined repositories. For each country, we searched: (i) official gazettes and national legal information systems; (ii) official websites of Ministries/Secretariats responsible for sport and disability; (iii) National Paralympic Committee websites (statutes, official guidance, program rules); and (iv) national sport institute repositories and national sport plans when applicable. Searches were conducted in Spanish, Portuguese, and English using consistent keyword combinations (e.g., “Paralympic” OR “parasport” OR “adapted sport” AND “law” OR “decree” OR “regulation” OR “statute” OR “plan” OR “program” OR “scholarship” OR “incentive” OR “accessibility” OR “classification” OR “eligibility”). Reference chaining was used to retrieve amendments, implementing decrees, and annexes explicitly cited within primary instruments.

Documents were eligible if they met at least one of the following criteria: (1) recognized the right to sport for persons with disabilities and/or defined adaptive/Paralympic sport; (2) established institutional arrangements (e.g., formal recognition and functions of the National Paralympic Committee; coordination with sport federations); (3) created national policy instruments relevant to Paralympic sport (e.g., scholarships/incentives, national plans, tax incentives); (4) published technical-sport protocols (classification/eligibility guidance, procedures, and/or calendars); or (5) established accessibility standards or equity measures (gender and/or territorial reach) applicable to the sport system. We excluded press releases without normative or official programmatic status, subnational documents without national effect, non-enacted bills, and repealed regulations without transitional effects.

### 3.2 Workflow, coding, and comparative procedure

The documentary workflow followed four stages: identification, screening, eligibility, and inclusion. After identification, duplicates were removed. Screening excluded documents that did not meet scope or status criteria. Eligibility assessment verified normative force (enacted law/decree/regulation) or official institutional issuance (e.g., formally published national plan or program rules) and checked current validity or transitional effects. Each included document was coded using a standardized extraction form capturing metadata (country, instrument type, title/identifier, issuing body, year, validity status, and URL) and substantive content relevant to the objectives (rights/definitions; governance arrangements; policy instruments; classification/eligibility provisions; pipeline measures; accessibility/equity).

Cross-country comparison was conducted using a country-by-dimension matrix built from the coded extraction forms. We compared countries horizontally within each dimension (to identify convergences and gaps) and vertically across dimensions within each country (to assess internal coherence of the policy package). Differences were evaluated using three predefined documentary anchors: explicitness (formal regulation vs. generic statements), operational specificity (presence of procedures, responsible bodies, eligibility rules, timelines, or monitoring requirements), and public visibility (availability of the supporting document in official open sources). When the manuscript describes systems as “more articulated” vs. “more fragmented,” these terms refer to these documentary anchors rather than to an external benchmark. Specifically, “more articulated” denotes cases where multiple dimensions are supported by publicly available instruments with defined procedures and responsible bodies, whereas “more fragmented” denotes cases where provisions remain generic and/or operational instruments are not publicly accessible.

In line with interpretive documentary analysis, empirical patterns and their interpretation are reported conjointly in the Results/Discussion section. This integrated presentation is intended to preserve traceability between the interpretive arguments and the policy instruments.

### 3.3 Methodological limitations

Comparability is conditioned by asymmetries in public documentation. Some countries do not publicly disseminate classification protocols or calendars; in those cases, the technical-sport dimension was coded conservatively based only on traceable official sources. We also note variability in the legal force of instruments (laws/decrees vs. plans/guidelines); this was addressed by documenting instrument status and prioritizing higher-ranking norms when inconsistencies occurred. Finally, the analysis is documentary by design and therefore evaluates what is formally established and publicly visible; implementation practices that are not documented in open sources may not be captured.

## 4 Results and discussion

Results are reported as document-grounded findings derived from the comparative extraction matrix and are presented together with their interpretation to preserve traceability between claims and sources. Specifically, we summarize what is explicitly established in national legal frameworks and official programmatic instruments regarding (i) definitions of disability and the formal inclusion of Paralympic/adaptive sport, (ii) incentives and support schemes for Paralympic athletes, and (iii) pipeline-related mechanisms (talent identification, school–club–national team transitions, and equity approaches). Interpretive concepts such as meritocracy, ableism, and structural inequality are therefore discussed alongside the corresponding documentary patterns, using the pre-specified evaluation anchors to support cross-country comparisons.

### 4.1 Definitions of disability and paralympic sport in each country's policies

The first point to be made is that an uneven historical trajectory is evident among the countries analyzed. Countries such as Brazil, Colombia, and Mexico incorporated adapted sport early into sectoral laws (2000–2005), whereas others did so later. Argentina did so in 2009 through ENARD, and Ecuador in 2010. Cuba recognized it from 1976 based on the universal principle of sport, but only in 2025 enacted a specific law with chapters that included explicit references to Paralympic sport. In most countries, the first policy was declarative, meaning the mention of sport for persons with disabilities in framework laws, whereas policy and programs concerning scholarships, high-performance plans, and Paralympic infrastructure emerged between 2005 and 2015.

This greater involvement may be associated with progress in the international normative framework on the rights of persons with disabilities and its ratification by the different countries. At the same time, the IPC Classification Code establishes the binding framework of the Paralympic Movement for sport-specific eligibility and refers to technical standards that take the ICF as a conceptual basis, requiring national systems to move from static notions of impairment toward functional and sport-specific criteria for each discipline.

Table 3 shows how each country has incorporated sport for persons with disabilities. It is important to recognize that countries have advanced through two and even three different laws in order to achieve alignment with the Convention.

In comparative terms, substantive convergences can be observed around definitions of disability in most countries. Chile, Peru, Costa Rica, Brazil, Mexico, and Ecuador have adopted definitions of disability compatible with the CRPD, that is, centered on the interaction between impairment and barriers, with recognition of the right to sport and recreation as part of the catalog of rights. In all of them, the entry point to Paralympic sport occurs through disability framework laws complemented by sport laws and policies, which facilitates articulation with the sport-specific classification required by the IPC.

The differences emerge mainly in the degree of detail with which Paralympic sport is recognized and in the technical depth of the regulation: Brazil and Colombia more clearly integrate the legal infrastructure needed to sustain processes of classification and participation by sport. Chile stands out for a legal definition of adapted sport consistent with Paralympic practice, whereas Mexico strengthens the technical link with the ICF through health certification. Argentina ensures the highest normative force by granting constitutional rank to the CRPD, although sport specificity is structured more through sectoral and federative regulations than through autonomous legal definitions. Cuba, for its part, now has an inclusive sport law of general scope that recognizes the Paralympic Committee, but the publication of detailed technical guidelines on classification and eligibility is still incipient. Finally, Venezuela maintains a more biomedical definition in its 2006 law, although its later sport framework has sought to move closer to the CRPD principles of equality and nondiscrimination. In summary, the tendency among the countries occupying the highest places in the Paralympic medal table is toward the interaction model of the CRPD and the progressive incorporation of the language and functional logic underlying the IPC Classification Code.

It is important to mention that most of the laws in which Paralympic sport is explicitly addressed in the countries analyzed date from after the year 2000, with the exception of Cuba and Peru. This points to a process of recognition that has been both slow and very recent, which may indicate that the regulations and protocols are still in the process of consolidation.

### 4.2 Instruments and incentives for paralympic athletes in the 10 countries

As research in the field clearly indicates, medal-table results are crucial for countries to support and sustain the development of Paralympic sports. Although all recognize the right to sport, the most funded instruments continue to be achievement-based scholarships and high-performance plans, with scant attention to early talent identification and mass participation, with the partial exceptions of Brazil, Chile, and, to a lesser extent, Colombia.

Scholarships for athletes with disabilities tend to be organized according to the logic of high performance and competitive merit, not according to a compensatory logic for structural inequality. This pattern is documented in the eligibility rules summarized in Table 3, where access is linked to sporting results, rankings, selection status, or placement within high-performance categories (e.g., Brazil's Bolsa Atleta, Law 10.891/2004; Chile's PRODDAR regulations; Peru's PAD categories; Argentina's ENARD scholarship rules). In practice, the system primarily recognizes Paralympic athletes once they have already entered the qualifying, selection, or podium circuits; therefore, scholarships function more as mechanisms to maintain performance than as tools to democratize access. This structure produces formal but selective inclusion: it better protects those who have already overcome barriers related to infrastructure, functional classification, training costs, and institutional mediation, which hinders early development in sport for many athletes with disabilities.

On the other hand, the stability of the scholarship depends on the state's ability to convert sporting achievement into a verifiable administrative category. Where more precise regulations exist, the scholarship defines time frames, grounds for retention, application windows, and amount hierarchies; where regulation is more general, support exists, but its concrete conditions remain less transparent. In this sense, Chile, Brazil, Argentina, Costa Rica, and Ecuador display more formalized designs; Colombia, Peru, and Mexico present clear support schemes but with greater dependence on calls, guidelines, or sectoral management; and in Cuba and Venezuela the framework reviewed makes it possible to affirm the existence of state support, although the operational conditions of the scholarship as a periodic benefit are not equally explicit in open official sources.

As shown in Table 4, scholarships for athletes with disabilities are not structured as a distinct inclusion policy, but rather as an extension of general high-performance regimes. This matters because it reveals the logic of conditional integration: persons with disabilities are incorporated to the extent that they meet performance thresholds defined by the high-performance system (e.g., ranked results, selection continuity, or podium outcomes as specified in Bolsa Atleta, PRODDAR, PAD, and ENARD rules). In this sense, access to economic support rests less on recognition of structural inequality and more on the ability to demonstrate results within a model that privileges competitiveness and excellence. The comparative corpus therefore supports the interpretation that inclusion is formally recognized but remains selective and hierarchical, consistent with meritocratic policy design.

The documentary corpus also shows that most systems operationalize support through an individualized understanding of sporting success: scholarships and incentives are granted on the basis of achievements, rankings, competitive continuity, or formal membership in high-performance programs (Table 4), while structural costs associated with disability (accessibility, transportation, specialized equipment, guides/assistants, and regional disparities) are addressed more unevenly and are often confined to non-monetary provisions when formally specified (Table 5). This documentary pattern provides the empirical basis for interpreting a structural inequality problem: policy intensity concentrates downstream (after selection/qualification), whereas upstream conditions for access and retention are less consistently formalized across countries.

With respect to medal awards, they follow the same logic as scholarships: rewarding attained performance and sustaining trajectories already legitimized by the competitive system. Medal awards are the most visible expression of this logic, because they concentrate the value of support on the final result and not on the process that precedes that achievement. Tax incentives and funding, for their part, strengthen the institutional structure of adapted sport, but they generally do so from a perspective of high-performance administration rather than from an explicit policy of social justice or barrier removal. Non-monetary support constitutes an interesting space for analysis. In this form of support, a conception of success in sport performance has materialized as attached not only to individual effort, but also to material, technical, medical, and relational conditions. When states include insurance, multidisciplinary teams, training centers, functional classification, support for guides, or child care, they move partially away from a strictly meritocratic view toward a more social understanding of disability. Even so, across the region there still predominates a policy that recognizes the athlete with a disability as an exceptional high-performance subject, rather than as a rights-bearing subject who requires stable structural conditions to participate on an equal basis.

### 4.3 Talent identification and equity in paralympic sport

Comparative evidence suggests that the inclusion of persons with disabilities in high-performance sport continues to be mediated by a tension between formal integration and structural inequality: although states increasingly recognize Paralympic sport in their normative and programmatic frameworks, not all of them develop, with equal clarity, mechanisms of early talent identification, bridges of continuity between levels, or explicit strategies to correct gender and territorial gaps.

The national cases analyzed show that the consolidation of Paralympic sport in these countries depends not only on the existence of economic support or recognition of performance, but also on state capacity to structure pathways of access, continuity, and expansion of opportunities. In this sense, the three dimensions examined in this section—talent identification, the transition between school, club, and national team, and gender- and territory-based approaches—make it possible to view Paralympic sport not only as a competitive space, but also as a field of institutional production of sport trajectories. Some countries have advanced toward more systematic models of identification and support, whereas others maintain fragmented or weakly formalized schemes in which access depends more on preexisting networks, territorial availability, or partial forms of articulation among agencies.

Regarding talent identification, Table 6 shows how this process is carried out in each country.

As documented in Table 6, some countries have moved toward institutionalized talent identification mechanisms, while others rely on fragmented or implicit devices. Brazil, Chile, and Ecuador provide the clearest documentary evidence of named identification schemes and implementing structures—respectively, Paradesporto Brasil em Rede (PPBR) linked to SNPAR; Promesas Chile (IND) complemented by COPACHI camps; and the High-Performance Plan (PAR 2022–2025) with categorization and quotas. By contrast, in Cuba and Venezuela the available legal and institutional documents recognize Paralympic structures but do not specify a publicly accessible, sport-specific identification protocol; in Peru and Costa Rica, identification appears dispersed across general sport frameworks or institutional follow-up documents without a single standardized parasport protocol (Table 6). These documentary differences substantiate that early-stage access is more policy-driven in some cases and more contingent on preexisting networks and territorial availability in others.

This reveals that “talent identification” is not a neutral process: it defines who becomes visible to the system and who is excluded even before competing. In Paralympic sport, this stage is traversed by barriers of accessibility, unequal schooling, availability of functional classification, and the urban concentration of resources. For that reason, countries that support identification through universities, specialized centers, or regional programs tend to build a more robust identification system; those that do not reproduce a selective inclusion in which only those who have already overcome considerable structural obstacles manage to enter.

Another aspect of the process that an athlete must navigate in order to become a Paralympic athlete concerns the transition between the school system, a club, and subsequently the national team. The regional comparison suggests that the transition between school, club, and national team remains one of the most unequal points in Paralympic sport. In several cases there is a relatively legible route, as in Brazil, Mexico, Chile, Argentina, or Ecuador, but in others the transition appears more generic or weakly protocolized, as occurs in Cuba and Venezuela. Moving from school to club and from there to the national team requires physical accessibility, functional classification, transportation, technical support, and institutional recognition of Paralympic bodies and modes of competition. Where the system does not produce clear bridges, persons with disabilities are exposed to early dropout, truncated trajectories, and an individual overload in sustaining their careers. In that sense, the more solid routes are also those that closely approximate the logic of rights, because they reduce dependence on isolated merit and strengthen conditions of continuity.

Finally, we examined whether gender- and territory-based gaps are addressed in the documentary corpus through binding targets or operational provisions. Table 8 shows that this component is the least consolidated across the ten countries: Brazil is the only case where the main identification instrument reviewed (PPBR) explicitly combines territorial expansion with participation targets by sex in its calls, while Chile and Costa Rica show partial developments through programmatic actions or sex-disaggregated reporting without comparable binding targets. In most other cases, equity is expressed as general inclusion language with no mandatory targets, monitoring indicators, or specific mechanisms to correct underrepresentation of women with disabilities or capital–periphery gaps (Table 8). This documentary pattern shows that equity remains declarative rather than operationalized within Paralympic policy in the region.

**Table 8:** Gender- and territory-based approaches to talent identification by country.

Country	Gender/territory approaches
Brazil	The gender and territory approach is formulated more explicitly through the Paradesporto Brasil em Rede program, which combines a logic of territorial expansion with criteria of equity in participation. The mechanism seeks to broaden coverage toward historically less favored regions, especially the North and Northeast, while incorporating participation targets by sex in its calls. This suggests a more active understanding of territorial and gender inequality within Paralympic policy.
Colombia	The territorial approach is articulated through the structure of the National Sport System, which organizes public action through an institutional network with nationwide reach. However, at the gender level no explicit targets or specific mechanisms for correcting gaps are identified, so treatment of this dimension remains more implicit than programmatically developed.
Mexico	Territorial and social inclusion is channeled mainly through specialized devices such as the Mexican Paralympic Center, which concentrates support and development functions. Nevertheless, the gender component does not appear standardized in homogeneous public targets, which suggests an approach oriented more toward general inclusion than toward a clearly defined intersectional policy.
Argentina	The gender approach appears through specific training and awareness actions within the sport field, although without a single, national, and binding target to structure Paralympic policy in this matter. In territorial terms, the logic continues to depend to a large extent on articulation among sport and institutional actors rather than on an explicit design of territorial redistribution.
Cuba	The normative framework expressly recognizes principles of accessibility, inclusion, and gender equity, which gives these dimensions a formal place within the sport system. However, the available information does not show public operational targets or specific implementation mechanisms, so the approach appears more as a guiding principle than as a verifiable programmatic device.
Venezuela	The gender approach is observed in fragmented form in some calls or actions differentiated by sex, but no public national guidelines are identified that structure this dimension in a sustained way. In territorial terms, functioning seems to rest more on the general organization of the sport system than on an explicit strategy to correct regional inequalities.
Costa Rica	The territorial approach appears implicitly through the cantonal scale and institutional follow-up mechanisms, while in gender there are reports that disaggregate information by sex. However, that disaggregation does not necessarily translate into binding targets or into a specific policy for promoting women with disabilities in Paralympic sport.
Chile	The mechanism combines a decentralized territorial logic, visible in the regional strategies developed by the Regional Ministerial Secretariats, with specific actions aimed at girls and women in the Promesas Chile program. Although this combination shows greater sensitivity to territorial and gender inequality, no single national target has been identified that articulates both dimensions in a binding way.
Peru	General guidelines of inclusion within sport predominate, without specific national gender targets being identified for the Paralympic sphere. This indicates that both the gender perspective and territoriality operate more as broad principles of public policy than as concrete mechanisms of redistribution or correction of inequalities.
Ecuador	The High-Performance Plan proposes a decentralized model that recognizes the territorial dimension in the organization of the sport system. Nevertheless, the public document does not make specific gender targets visible, so the policy appears to advance more in territorial decentralization than in the development of an explicit intersectional perspective.

A policy that incorporates no targets, monitoring, or specific actions runs the risk of universalizing an abstract subject of adapted sport, making invisible the fact that not all persons with disabilities face the same barriers to entering, training, and remaining. The absence of a robust intersectional perspective reproduces a hierarchical inclusion: those who live near the centers, have family or institutional mediation, and better fit the historically dominant profile of high performance enter more easily. For that reason, gender and territory are not complementary variables, but structural dimensions for evaluating justice in Paralympic talent identification.

The joint analysis of talent identification, school-club-national team transition, and the gender/territory approach shows that the region presents highly unequal levels of institutionalization of Paralympic sport. The most advanced cases are Brazil, Chile, and Ecuador, because they have relatively identifiable devices for identifying athletes, organizing their competitive progression, and sustaining that trajectory within national high-performance programs. Brazil stands out for articulating identification, transition, and a more explicit formulation of territorial equity and participation by sex; Chile stands out for the clarity of the route from the school and municipal base toward the national team, in addition to incorporating regional actions and some initiatives with girls and women; Ecuador shows strength in the integration among federations, the Paralympic Committee, and the High Performance Plan, with a more stable structure of classification and follow-up. Mexico and Argentina are situated at a second level, presenting important mechanisms of development and support, but with less explicitness in the territorial and gender approach. Colombia has advanced in institutional reorganization by sport rather than by type of disability, which is important for competitive specialization, although it still maintains gaps in the explicit formulation of gender targets and in the visibility of its identification routes.

By contrast, Cuba, Venezuela, Costa Rica, and Peru show partial or less systematic frameworks: sport structures, general guidelines, or sectoral experiences exist, but clear and differentiated public protocols for Paralympic sport do not always appear in these three dimensions. From a public policy perspective, the main regional challenge is to move from declarative inclusion to structured inclusion, that is, to build systems that do not depend on individual effort, family networks, or the urban concentration of opportunities.

## 5 Conclusion

This study set out to conduct a comparative analysis of public policies for the promotion of Paralympic sports in ten Latin American countries, focusing on three dimensions: regulatory frameworks, operational mechanisms, and equity criteria. Based on the review conducted, the main conclusion is that Latin America has made significant progress in the formal recognition of the right to sports for persons with disabilities. This progress accelerated in the period following the adoption of the Convention on the Rights of Persons with Disabilities and its gradual ratification across the region (2007–2013), although ratification timelines varied substantially across countries. However, this regulatory progress does not uniformly translate into robust institutional frameworks capable of guaranteeing access, continuity, and the advancement of sports.

The comparison shows that the countries with the best results are not necessarily those that simply have more laws, but rather those that have successfully integrated laws, incentives, technical support, and institutional pathways to promote sports development. Brazil stands out as the most well-established case, combining a regulatory framework aligned with the Convention, formalized economic incentives, non-monetary support, talent identification, and a degree of clarity regarding territorial and gender criteria. Chile and Ecuador also show significant progress due to the existence of clearer pathways between grassroots training, development programs, and high performance. Mexico and Argentina occupy an intermediate position, with some funding and support mechanisms, though with less clarity in certain aspects of equity. On the other hand, in countries such as Cuba, Venezuela, Costa Rica, and Peru, higher levels of fragmentation or fewer published protocols persist, particularly regarding identification, transition, and follow-up criteria.

This study confirms that regional policy is organized primarily around high performance and rewards based on competitive merit. Across the documentary corpus, the main scholarship and incentive schemes are triggered after athletes enter ranked competition, selection systems, or podium outcomes (Table 4), as reflected in eligibility rules tied to results or high-performance categories (e.g., Bolsa Atleta categories; PRODDAR categories; PAD DC/DCAN tiers; ENARD merit criteria). This means that state intervention is most intense at the stage of established performance, whereas earlier phases of entry and retention rely more heavily on uneven local capacity, family resources, and the availability of classification, equipment, and accessible training environments.

Overcoming the gaps identified in this study requires moving away from an exclusive reliance on the medal count as an indicator of success and toward a broader conception of an athlete's performance. Only when public policies cease to be based on individual heroism and family sacrifice, begin to value sports practiced by persons with disabilities, and assume the duty to actively redistribute conditions of access and continuity, will it be possible to speak of a more just, sustainable, and rights-respecting regional Paralympic system in the region.

Policy implications point to three concrete levers for strengthening professionalization in adaptive and Paralympic sport systems. First, complement performance incentives with stable support for access and continuity (transport, equipment pathways, and entry-level training support). Second, publish and standardize sport-specific procedures and calendars (including classification/eligibility pathways and progression criteria) to reduce uncertainty and improve transparency. Third, secure continuity of coaching and integrated support teams through multi-year program rules, and embed measurable equity targets (gender and territorial reach) with monitoring and public reporting.

## Data Availability

The original contributions presented in this work are included in the article/Supplementary Material, further inquiries can be directed to the corresponding author.
